# Letter to: “High purpose in life of older adults is associated with younger age, higher education, and being female”: methodological and analytical considerations

**DOI:** 10.1055/s-0046-1829019

**Published:** 2026-11-03

**Authors:** Himanshu Jaiswal, Akanksha Saxena

**Affiliations:** 1 Maharishi Markandeshwar (Deemed to be University), Maharishi Markandeshwar Institute of Physiotherapy & Rehabilitation, Mullana, Ambala HRIndia.

Dear Editor,

We read with interest the article by Silva et al. 1 examining the associations involving sociodemographic variables and purpose in life (PiL) among cognitively-healthy older adults in Brazil. The authors should be commended for addressing an important yet underexamined dimension of psychological wellbeing in later life within a Brazilian context where evidence on PiL remains limited. Their findings, suggesting associations of higher PiL with higher level of schooling and the female sex, contribute meaningfully to discussions on psychosocial determinants of healthy aging. At the same time, several methodological and statistical considerations may help contextualize these findings.

A key methodological issue relates to the study population. Although presented as cross-sectional, the participants were drawn from the 24-month follow-up of a randomized cognitive-stimulation trial. 1 Individuals retained in long-term intervention studies often represent a healthier, more motivated, and socially-active subgroup, introducing selection and retention bias. Moreover, prior exposure to cognitive stimulation may itself influence psychosocial constructs, including meaning, engagement, and life purpose, even when PiL was not the intervention target. Adjustment for original trial allocation, or sensitivity analyses stratified by treatment arm, would have strengthened internal validity, as recommended in methodological guidance on secondary analyses from randomized cohorts. 2

A second issue is the dichotomization of PiL using a median split before the regression analysis. While convenient, converting a continuous psychological construct into a binary outcome reduces statistical power, obscures within-group variation, and may create artificial distinctions between participants with similar scores. 3 Individuals scoring just above and below the median were assigned to different groups, despite likely having comparable levels of life purpose. Retaining PiL as a continuous outcome and examining associations using multivariable linear regression or generalized linear modeling would preserve information and produce more stable estimates. 3

The multivariable modeling strategy also warrants consideration. Stepwise logistic regression remains controversial because automated variable selection may generate unstable models, increase spurious findings, and produce coefficients overly dependent on a single sample. 4 A stronger approach would be a prespecified multivariable model informed by theoretical and empirical evidence, including variables known to influence PiL, alongside reporting of goodness-of-fit, explained variance, calibration, and multicollinearity. These steps improve transparency and reproducibility in observational modeling. 5

Interpretation would also benefit from closer attention to effect size. Correlations between PiL and age (rho = -0.151) and schooling (rho = 0.156) were statistically significant but weak, explaining only a small proportion of variability in PiL. Likewise, the regression estimate for schooling (OR = 1.087 per additional year) reflects only a modest incremental association. Statistical significance should therefore be interpreted alongside practical significance, particularly given the likely residual confounding. Important determinants of PiL, including physical health, functional capacity, social connectedness, loneliness, religiosity, income, and subclinical depressive symptoms, were not included, despite strong evidence linking these factors with life purpose in older adulthood. 6

Measurement methodology introduces another limitation. The Purpose in Life Scale was originally validated through direct administration, whereas Silvs et al. ^1^ administered it by telephone. Changes in administration mode can affect comprehension, response behavior, and psychometric performance, particularly among older adults. 7 Reporting internal consistency estimates for telephone administration, or validation evidence supporting remote use, would strengthen confidence in measurement reliability.

Finally, there appears to be a discrepancy between the article title and the multivariable findings. The title suggests that high PiL is associated with younger age, higher levels of schooling, and being female, whereas age did not remain independently associated with PiL in the final regression model. 1 A more precise title reflecting independent multivariable associations would better align with the final analytical results and reduce overinterpretation.

Overall, Silva et al. 1 make a meaningful contribution by highlighting PiL as an important dimension of aging research in Brazil. Clarifying these methodological and analytical issues would further strengthen the interpretive value and scientific contribution of this work.
